# Race by Education Intersectional Differences in Exposure to Tobacco Advertisement in Baltimore City

**DOI:** 10.29245/2689-999x/2023/2.1184

**Published:** 2023-12-07

**Authors:** Payam Sheikhattari, Rifath Ara Alam Barsha, Jummai Apata, Shervin Assari

**Affiliations:** 1 Center for Urban Health Disparities Research and Innovation, Morgan State University, Baltimore, MD, USA; 2 The Prevention Sciences Research Center, School of Community Health and Policy, Morgan State University, Baltimore, MD, USA; 3 Department of Family Medicine, Charles R Drew University of Medicine and Science, Los Angeles, CA, USA; 4 Department of Urban Public Health, Charles R Drew University of Medicine and Science, Los Angeles, CA, USA

**Keywords:** Population groups, Tobacco advertisement exposure, Educational attainment, Intersectionality, Social determinants of health

## Abstract

**Objective:**

This study aimed to examine the intersectional effects of race and educational attainment on tobacco advertising exposure among adults in Baltimore, given the growing evidence on differential influence of education for Black and White populations.

**Methods:**

A survey was conducted in Baltimore, collecting data on educational attainment, demographics, and tobacco advertising exposure among adults (n = 3028, 22.7% 18 – 29, 17.9% 30 – 39, 23.4% 40 – 49, 20.9% 50–59, and 11.1% 60+ years old). The sample included both Black and White adult individuals. Logistic regression analyses were employed to assess the association between educational attainment and tobacco advertising exposure, without and with interaction with race, adjusting for relevant covariates such as age, gender, and employment. Sensitivity analysis also controlled for smoking status.

**Results:**

The study results indicated that while high educational attainment is associated with less exposure to tobacco ads, highly educated Black adults report significantly higher tobacco advertising exposure compared to highly educated White adults. Same results were observed after controlling for smoking status.

**Conclusion:**

Educational attainment may not exhibit a large protective effect against environmental risks such as tobacco ad exposure for Black populations, possibly because of segregation and racism that hinder highly educated Black people ability to move to low-risk neighborhoods.

## Introduction

Tobacco use and its consequences have long been recognized as a public health concern, with a disproportionate impact on Black communities^1,2^. Despite lower prevalence of tobacco use within these communities (for example 13.5% and 16% in Black and White adults according to the American Lung Association)^3^, various factors^4^ such as limited access to cessation programs, comorbid conditions, the preference for menthol cigarettes contribute to higher tobacco-related consequences in such populations^5^. It is imperative to gain a comprehensive understanding of this issue in order to develop effective tobacco policies and interventions.

Recent research on the Minority Diminished Returns (MDRs) theory^6^ has shed light on the persistence of tobacco use among the Black middle class, which includes individuals with high levels of education^7–10^. Surprisingly, even children from highly educated racial and ethnic minority families have been found to be more likely to smoke^10^. Exploring the reasons behind the elevated risk of tobacco use among educated Black individuals, one study proposes that place plays a crucial role^11^. Specifically, it suggests that despite higher incomes and education levels, Black families are more likely to reside in disadvantaged areas with tobacco ad exposure compared to their White counterparts with comparable socioeconomic status^11^.

MDRs theory posits that due to racism, social stratification, and various obstacles, resources at the individual level are insufficient to generate equitable outcomes for racial and ethnic minorities^6^. Structural inequalities, such as segregation and associated environmental risks, further exacerbate the risk of tobacco use among the Black population, regardless of their educational or income levels^7–10^. The pervasiveness of structural, systemic, and interpersonal discrimination and racism places Black individuals at risk across different socioeconomic strata.

The impact of place on the increased prevalence of tobacco use within Black communities extends to tobacco advertising^11^. Point-of-sale advertisements, corner stores, and gas station promotions disproportionately target and expose Black communities to tobacco advertising^12,13^. Predatory marketing strategies employed by tobacco companies often specifically target urban areas with higher Black populations^13^.

A recent study examining MDRs found that highly educated young Black adults reported significantly higher prevalence of tobacco advertising exposure compared to their White counterparts, whereas highly educated young White adults reported very low prevalence^11^. This study, although limited to young adults, suggested that proximity to liquor stores and corner stores, that have point-of sale tobacco ads, may be a contributing factor, undermining the protective effects of education against tobacco advertising exposure for Black communities and individuals^11^.

To replicate and expand upon this national study, we conducted a survey in Baltimore that aimed to test the association between educational attainment and tobacco advertising exposure among adults. Our primary hypothesis was that higher educational attainment would be associated with lower odds of tobacco advertising exposure within the overall sample, comprising both White and Black individuals. Additionally, we hypothesized that the inverse association (i.e., protection) between educational attainment and odds of tobacco advertising exposure would be weaker for Black adults compared to their White counterparts. We also tested the same question after controlling for smoking status. By examining these associations at the local level and among a broader sample of adults, we aimed to contribute to the understanding of the complex dynamics underlying the relationship between educational attainment, place, and tobacco advertising exposure. The findings from this study have important implications for the development of targeted tobacco control policies and interventions to address the persistent disparities in tobacco-related consequences experienced by Black communities.

## Methods

### Study Setting and Sample

Communities Engaged and Advocating for a Smoke-Free Environment (CEASE)^14–16^ is a Morgan State University’s’ tobacco cessation program operating since 2008 to address tobacco related health disparities in Baltimore City through a community based participatory research (CBPR)^17,18^ approach. CEASE conducted a community survey in 2012–2013 as part of a project to better understand underserved community’s need related to tobacco. Adults aged 18 years and older were surveyed (n= 3,931) through a paper-based and self-administered questionnaire regardless of their smoking status.

### Analytical Sample

For this current study, only Black and White participants were included in the data analysis. In addition, participants with missing values were excluded and the final analytical sample consisted of 3,028 Black and White adults.

### Ethical Consideration

Informed written consent was obtained from all participants and the study was approved by MSU’s Institutional Review Board (IRB). To maximize the confidentiality of the participants, no personal identifier was used during the data analysis process. Project IRB #08/04–0023 & 11/02–0011. Approval Date: 2012.

### Measures

#### Outcome variables

Exposure to tobacco advertisement is the outcome variable. Participants were asked, “Are there billboards with tobacco ads in your neighborhood?” and “Do the local convenience stores advertise cigarettes in the store windows?”. Those who responded yes to any of these two questions were considered to be exposed to tobacco advertisement. The outcomes were operationalized as binary outcomes (No=0, Yes=1).

#### Independent Variables

##### Educational Attainment:

Education is the primary independent variable in this study. The educational level was categorized as (1) Some high school or less, (2) Graduated from high school, (3) One or more years of college, (4) Graduated from trade school (trains electricians, plumbers, and mechanics and other technicians), and (5) Graduated from college.

##### Employment:

Employment status is another independent variable and were operationalized as a categorical variable (No=0, Yes=1).

##### Demographic Covariates:

The covariates were age and gender when adjusting for confounding. Age was a categorical variable (18–29 years, 30–39 years, 40–49 years, 60 years & more), and gender was a dichotomous variable (women =0 and men =1).

##### Moderator:

Race was used as a moderating variable and was self-identified.

### Statistical Analysis

Data analysis was performed using Stata 15.0 (Stata Corp, College Station, TX: StataCorp LLC.). For univariate analyses, we presented the results in frequencies and percentages for categorical variables. For bivariate analyses, we used Pearson Chi-square tests. Four logistic regression models were estimated for our multivariable analysis. The first two logistic regressions were estimated in pooled samples. Model 1 was the main effect model without any interaction term. The race-by-SES indicator (educational attainment or employment) interactions were estimated in Model 2. Model 3 was estimated for Black adults and Model 4 for White adults. The stratified models were estimated to understand whether covariates acted differently across groups. We ran sensitivity analysis with smoking as a covariate and also smoking as strata. The regression results were presented as adjusted odds ratios (ORs) and 95% confidence intervals (CIs). The significance level was set as p <0.05.

## Results

This study included 3,028 adults who were either Black (n=2,134; 70.5%) or White (n = 894; 29.5%). Table 1 provides descriptive statistics of the study variables in the overall sample and by race. Only 13.6% (n = 290) of Black adults were college graduates in our sample compared to 45% (n = 402) of White adults. About 48.4% (n = 1033) of Black adults were unemployed compared to 35.1% (n = 314) of White adults. The exposure to tobacco advertisement was high among all participants, and the rate was similar for Black and White adults (94.8% vs 89.9%; n = 2023 and 804 respectively). About 56.5% (n = 1206) of Black adults reported being current smokers compared to 37.2% (n = 333) of White adults.

Table 2 describes the prevalence of tobacco exposure across race and educational attainment intersectional groups. The tobacco advertisement exposure among low educated Black (less than high school) individuals was 94.5% (n = 514). Highly educated Black (graduated from college) individuals had 90.0% (n = 261) exposure to tobacco advertisement. Whites who had less than high school diploma had the highest percentage of exposure (n = 140; 97.9%). The exposure among White highly educated (college graduation) individuals was 84.3% (n = 339).

Table 3 presents the results of four logistic regression models. While *Model* 1 only included the main effects of education, employment, and race, *Model 2* also included interaction between education and race and employment and race. Based on *Model 1*, there was a significant association between higher education and exposure to tobacco advertisements. College graduates were significantly less likely to be exposed to tobacco advertisements than those who attended some high school or less (Adjusted OR: 0.39, 95% CI: 0.24–0.62). In addition, employed adults were less likely to be exposed to tobacco advertisement; however, the result was not significant. A significant interaction between race and education on exposure to tobacco advertisement was observed in *Model 2*. Based on Model 2, the protective effects of education on tobacco advertisement exposure were larger for White adults than Black adults (Adjusted OR: 5.48, 95%CI: 1.43–21.03). No significant result was found between employment and race interaction. *Model 3* showed that high education such as college graduates (Adjusted OR: 0.58, 95% CI: 0.32–1.05) and being employed (Adjusted OR: 0.90, 95% CI: 0.52–1.56) were associated with lower odds of exposure to tobacco advertisements for Black adults. Model 4 also showed a significant protective effect of education on exposure to tobacco advertisements for White adults (Adjusted OR: 0.10, 95% CI: 0.03–0.36). In addition, employed White individuals were less likely to be exposed to tobacco advertisement. However, the result was not significant.

The bivariate association between tobacco advertisement exposure and lifetime smoking overall was (OR =2.09; 95% CI: 1.34–3.24) (Table 4). For Black individuals (Model 3), the odds of tobacco exposure were significantly higher among ever-smokers (OR = 2.02; 95% CI: 1.08–3.76); however, the association was not significant for current smokers. For those who are Whites (Model 4), the odds of tobacco exposure were significantly higher among both ever smokers (OR = 2.46; 95% CI: 1.30–4.63) and current smokers (OR = 3.86; 95% CI: 1.31–11.39).

Table 5 shows the four logistic regression models. While Model 1 and Model 2 were performed in the pooled sample, Model 3 was performed for those who were ever smokers, and Model 4 was performed for those who were never smokers. Model 2, Model 3, and Model 4 included interaction terms. Higher education, such as college graduation, was associated with lower odds of exposure to tobacco advertisement for both ever smokers (OR= 0.12; 95% CI: 0.01–1.06) and never smokers (OR = 0.25; 95% CI: 0.05–1.17); however, the associations were not significant. Though results showed that employed and ever smoker people were less likely to be exposed to tobacco advertisement (OR = 0.95; 95% CI: 0.01–1.06) and employed and never-smoker people were more likely to be exposed to tobacco advertisement (OR = 1.03; 95% CI: 0.53–1.97), the associations were not significant. For both current smokers and never smokers, the odds of exposure to tobacco advertisement were higher for Black individuals with a college degree compared to white individuals with same education level; however, the findings were not significant.

## Discussion

The findings from this local sample in Baltimore reveal that while higher educational attainment was associated with lower odds of tobacco advertising exposure overall, this association varied by race and was weaker for Black individuals compared to White individuals. The result did not change without and with smoking as a covariate. This indicates that highly educated Black individuals remain at risk of tobacco advertising exposure, compared to their highly educated White counterparts.

To help better understand our results, while for Whites, prevalence of tobacco ad exposure was 97.9, 94.6, 90.3, 93.6, and 84.3, respectively across levels of education as education increase, these rates were 94.5, 96.0, 95.3, 97.9, 90.0 for Black adults. That is for example 13.6% and 4.5% difference in tobacco ad exposure between lowest to highest education in White and Black adults respectively.

These findings align with a previous study that used the Population Assessment of Tobacco and Health (PATH) data and reported similar results among highly educated young adults^11^. Specifically, highly educated young Black adults exhibited significantly higher prevalence of tobacco advertising exposure compared to highly educated young White adults, who reported very low prevalence^11^. It is hypothesized that proximity to liquor stores and corner stores may undermine the protective effects of education against tobacco advertising exposure for Black communities and individuals^11^.

The main effect of socioeconomic status indicators such as education on tobacco advertising has been documented in several studies. Barbeau et al found that fewer tobacco advertisements were located in higher socioeconomic communities, compared to the lower socioeconomic communities with three times as many brand advertisements as youth access signs in the lower socioeconomic communities^19^. Another study by Nian et al showed a higher level of targeting of individuals from low-SES neighborhoods by tobacco industry through several advertising channels^20^. Other studies have reported varying levels of tobacco advertising exposure and related tobacco use behavior by socioeconomic status^21,22^. These studies all highlight the protective effect of higher socioeconomic status, which is a reflection of higher educational attainment, on exposure to tobacco advertisement.

The only similar study that is available for comparison is the one by Assari in 2020. The study used PATH data and tested the association of educational attainment and race/ethnicity with exposure to tobacco advertisement among US young adults. Although tobacco ad exposure was lower in the national level than Baltimore, SES was lower in Baltimore, and place was mainly constrained in Baltimore study, we found similar results in Baltimore and US as a whole. So, both in a community survey in 2012–2013 in Baltimore City that does not use a random sample, and in 2016 random sample of young adults in the US, highly educated Back people remain at risk of exposure to tobacco ad exposure. This is another indication suggesting that MDRs hold regardles of setting, sampling, study design, or even cohort. The unique contribution of this article is replication in the local setting in more an empowered area.

In addition to socioeconomic status, some studies have documented the association between race/ethnicity and tobacco product advertising. Black people were found to be exposed to a higher concentration and density of pro-tobacco advertising compared to their Caucasian counterparts^23^. Moran et al observed that exposure to cigarette and non-large cigar advertising, as well as tobacco product promotions such as coupons, sweepstakes and free samples varied by ethnicity and socioeconomic level, with Black people and individuals of lower socioeconomic having the highest exposures^24^.

These disparities in exposure to tobacco advertising by race or socioeconomic status point to the selective targeting of vulnerable populations by the tobacco industry. Point-of-sale advertisements, corner stores, and gas station promotions have been shown to disproportionately target and expose Black communities to tobacco advertising. The tobacco industry has systematically and selectively targeted people of color, people with lower socioeconomic status, education, sexual minority groups and youth over the years^25–27^ with a resultant increase in tobacco use by the targeted populations in the target communities^20^. A literature review by Cruz et al. identified more studies of pro-tobacco marketing with planned efforts to increase tobacco use, such as greater density of tobacco billboards or promotions in retail outlets, in predominantly Black communities compared to studies on anti-tobacco campaigns^28^. Another systematic review found more menthol flavored tobacco marketing targeting urban neighborhoods and neighborhoods with more Black residents compared to rural neighborhoods and neighborhoods with more White residents^2^.

Educational attainment may not provide a significant protective effect against environmental risks for Black populations because of the pervasive influence of structural and interpersonal racism. A study developed a measure of structural racism at the county level, considering factors such as racial segregation, incarceration, educational attainment, employment, and economic status/wealth. They revealed significant geographic differences in the levels of structural racism, with higher levels generally observed in the Midwest and Northeast^29^. Another study discussed how systemic racism extends beyond the justice system to social, environmental, and economic structures, affecting health outcomes. They highlight how segregation, enforced by federal policies, results in limited employment opportunities, poor access to healthcare, low educational attainment, and low socioeconomic status for underrepresented minorities^30^. In another study, in 2014, Buot and colleagues examined the relationship between numerous social indicators and health across eighty large U.S. cities^31^. They found that health among black individuals correlated significantly with numerous economic factors such as segregation. They further argued argument that environmental risks for Black populations are influenced by a complex interplay of factors, including structural racism, that cannot be fully mitigated by educational attainment alone. Another study in 2021 delved into the issue of anti-Black racism and student discipline in schools, examining the perception, experiences, and alternatives of zero-tolerance policies in education^32^. They proposed interventions that align with the call to action by Black Lives Matter at Schools, emphasizing the need to end practices that contribute to the school-to-prison pipeline and perpetuate racial inequality. In 2016, Da Costa underscored the role of black political struggle in shaping anti-racist education policy. All these papers suggest that educational attainment alone may not be sufficient to mitigate environmental risks for Black populations, as these risks are intertwined with broader systemic issues^33^.

Future research should investigate the extent to which place plays a role in the increased prevalence of tobacco use within Black communities, extending to tobacco advertising exposure. Future studies should test if any proxy of place, characterized by the proximity to liquor stores and corner stores, diminishes the protective association between educational attainment and tobacco advertising exposure, particularly among Black adults. Structural inequalities, such as segregation and discriminatory marketing practices, contribute to the heightened risk of tobacco advertising exposure in Black communities, even among highly educated individuals. Future research should investigate the role of structural racism and segregation in reducing the protective effects of education for Black people. Similarly, studies should test the role of place-based factors that alter tobacco-related disparities in highly educated Black people.

It is essential to acknowledge the limitations of this study. The small sample size, non-random sampling, focus on Black and White populations, and reliance on self-reported data constrain the generalizability of the findings. Future validation studies could incorporate biomarker data, such as nicotine levels in urine, to supplement self-reported data. Additionally, the inclusion of confounders such as income and neighborhood socioeconomic status would provide a more comprehensive analysis. Despite these limitations, this pilot study contributes to the field by replicating a national finding within a local context, suggesting that the patterns observed among highly educated young Black adults across the US may also apply to the Black population in Baltimore.

Education alone is not a panacea for addressing inequalities. In fact, education can sometimes perpetuate existing racial disparities^34^. Lower quality of education in predominantly Black neighborhoods, preferential hiring and promotion of White individuals with similar education, and the concentration of better job opportunities in proximity to White populations can contribute to better health of highly educated White than Black people, so racial inequalities would sustain and may even widen in highly educated people^35^. Furthermore, highly educated Black individuals residing in disadvantaged areas face additional challenges related to segregation and limited opportunities for upward mobility^36^. According to some studies, SES may increase exposure and susceptibility to discrimination for Black people^37–40^, and we know that people may use tobacco and other substances to cope with their discrimination exposure^41^. Therefore, policies focused solely on education may fall short unless other factors, such as educational quality, labor market discrimination, and job availability, are also addressed.

We need to emphasize that this survey was conducted in 2012–2013, a decade ago. Over this period, the tobacco landscape has witnessed substantial changes, impacting advertising practices. Given this temporal shift, it is crucial to investigate whether new policies regarding advertisements have been implemented in Baltimore during this timeframe. Tobacco and social policies could be pertinent to the results. We acknowledge this temporal gap as a limitation of this study.

In conclusion, contrary to the traditionally held belief that education may be the solution for racial inequalities in the US, educational attainment may not exert similarly powerful protective effect against environmental risks for Black and White populations, possibly due to the enduring impact of segregation and racism that have historically hindered Black communities, families, and individuals across various levels of educational attainment. The pilot nature of this study highlights the need for further research to delve deeper into this topic and uncover additional insights into the complex interplay between education, place, and tobacco advertising exposure.

## Figures and Tables

**Table 1:** Descriptive statistics in the overall sample and by race

Variables	All (n=3,028)	Black (n=2,134)	White (n=894)
	N (%)	N (%)	N (%)
**Education** ***			
Some high school or less	687 (22.7)	544 (25.5)	143 (16.0)
Graduated from high school	1,024 (33.8)	819 (38.4)	205 (22.9)
One or more years of college	452 (14.9)	339 (15.9)	113 (12.6)
Graduated from trade school	173 (5.7)	142 (6.7)	31 (3.5)
Graduated from college	692 (23.9)	290 (13.6)	402 (45.0)
**Age (years)** ***			
18 – 29	809 (22.7)	486 (22.8)	323 (36.1)
30 – 39	542 (17.9)	366 (17.2)	176 (19.7)
40 – 49	709 (23.4)	554 (26.0)	155 (17.3)
50 – 59	631 (20.9)	495 (23.2)	136 (15.2)
60 & more	337 (11.1)	233 (10.9)	104 (11.6)
**Gender**			
Women	1,488 (49.1)	1,032 (48.4)	456 (51.0)
Men	1,540 (50.9)	1,102 (51.6)	438 (49.0)
**Employment** ***			
No	1,347 (44.5)	1,033 (48.4)	314 (35.1)
Yes	1,681 (55.5)	1,101 (51.6)	580 (64.9)
**Current Smoker** ***			
No	1,489 (49.2)	928 (943.5)	561(62.8)
Yes	1,539 (50.8)	1,206 (56.5)	333 (37.2)
**Exposure to Tobacco Advertisement** ***			
No	201 (6.6)	111 (5.2)	90 (10.1)
Yes	2,827 (93.4)	2,023 (94.8)	804 (89.9)

* *p*<0.05

** *p*<0.01

*** *p*<0.001 for comparison of Black and White adults

**Table 2:** Prevalence of tobacco ad exposure across race and educational attainment intersectional groups

Education	Exposure to Tobacco Advertisement	
	Black n (%)	White n (%)
Some high school or less	514 (94.5)	140 (97.9)
Graduated from high school	786 (96.0)	194 (94.6)
One or more years of college	323 (95.3)	102 (90.3)
Graduated from trade school	139 (97.9)	29 (93.6)
Graduated from college	261 (90.0)	339 (84.3)

**Table 3:** Logistic regression on the association between race, education, and employment with exposure to tobacco advertisement

Variables	All (n=3,028)		Black (n=2,134)	White (n=894)
	Model 1	Model 2	Model 3	Model 4
	AOR (95% CI)	AOR (95% CI)	AOR (95% CI)	AOR (95% CI)
**Education**				
Graduated from high school	1.13 (0.70–1.80)	0.36 (0.10–1.33)	1.42 (0.85–2.40)	0.36 (0.09–1.32)
One or more years of college	0.90 (0.52–1.56)	0.21* (0.06–0.79)	1.34 (0.70–2.57)	0.21* (0.06–0.80)
Graduated from trade school	1.76 (0.67–4.62)	0.31 (0.05–2.00)	2.87 (0.85–9.66)	0.30 (0.05–1.95)
Graduated from college	0.39*** (0.24–0.62)	0.11*** (0.03–0.36)	0.58 (0.32–1.05)	0.10*** (0.03–0.36)
Employment (Yes)	0.91 (0.65–1.27)	0.87 (0.51–1.50)	0.89 (0.58–1.38)	0.90 (0.52–1.56)
**Age**				
30 – 39	0.66 (0.43–1.02)	0.64* (0.42–0.99)	0.89 (0.47–1.68)	0.47* (0.26–0.86)
40 – 49	1.04 (0.64–1.70)	1.02 (0.63–1.68)	1.20 (0.63–2.29)	0.87 (0.40–1.90)
50 – 59	0.62* (0.39–0.97)	0.60* (0.38–0.95)	0.64 (0.36–1.15)	0.64 (0.30–1.37)
60 & more	0.33*** (0.21–0.52)	0.32*** (0.20–0.51)	0.36** (0.19–0.67)	0.30** (0.15–0.59)
Gender (Men)	1.94*** (1.42–2.64)	1.99*** (1.46–2.72)	1.85** (1.22–2.79)	2.23** (1.38–3.63)
Race (Black)	1.44* (1.05–1.98)	0.32 (0.09–1.11)	NA	NA
Race*Education				
Graduated from high school	NA	3.93 (0.96–16.02)	NA	NA
One or more years of college	NA	6.36* (1.45–27.81)	NA	NA
Graduated from trade school	NA	9.20 (1.00–84.58)	NA	NA
Graduated from college	NA	5.48* (1.43–21.03)	NA	NA
Race (Black)*Employment (Yes)	NA	1.05 (0.53–2.09)	NA	NA

Significance

* *p*<0.05

** *p*<0.01

*** *p*<0.001

AOR stands for Adjusted Odds Ratio

**Table 4:** Logistic regression on the association between race, education, and employment with exposure to tobacco advertisement with tobacco use as a covariate

Variables	All (n=3,028)		Black (n=2,134)	White (n=894)
	Model 1	Model 2	Model 3	Model 4
	AOR (95% CI)	AOR (95% CI)	AOR (95% CI)	AOR (95% CI)
**Education**				
Graduated from high school	1.27 (0.78–2.05)	0.43 (0.11–1.61)	1.52 (0.90–2.56)	0.48 (0.12–1.82)
One or more years of college	1.16 (0.78–2.05)	0.26* (0.07–0.99)	1.65 (0.85–3.22)	0.29 (0.07–1.16)
Graduated from trade school	2.05 (0.77–5.44)	0.33 (0.05–2.18)	3.28 (0.97–11.13)	0.33 (0.05–2.28)
Graduated from college	0.62 (0.38–1.02)	0.17** (0.05–0.60)	0.84 (0.45–1.54)	0.22* (0.06–0.78)
Employment (Yes)	1.07 (0.75–1.52	0.98 (0.56–1.69)	1.06 (0.67–1.66)	1.06 (0.59–1.88)
Ever smoker (Yes)	2.09** (1.34–3.24)	2.19** (1.41–3.41)	2.02* (1.08–3.76)	2.46** (1.30–4.63)
Current smoker (Yes)	2.07** (1.23–3.49)	2.01** (1.19–3.40)	1.76 (0.92–3.38)	3.86* (1.31–11.39)
**Age**				
30 – 39	0.56* (0.36–0.87)	0.54** (0.34–0.84)	0.76 (0.40–1.43)	0.37** (0.20–0.69)
40 – 49	0.77 (0.47–1.27)	0.75 (0.45–1.24)	0.89 (0.47–1.72)	0.59 (0.26–1.34)
50 – 59	0.43*** (0.27–0.68)	0.41*** (0.26–0.66)	0.42** (0.23–0.78)	0.49 (0.22–1.07)
60 & more	0.29*** (0.18–0.46)	0.28*** (0.17–0.45)	0.29*** (0.15–0.56)	0.27*** (0.13–0.56)
Gender (Men)	1.62** (1.18–2.23)	1.66** (1.21–2.29)	1.55* (1.01–2.36)	1.86* (1.13–3.06)
Race (Black)	1.41* (1.02–1.94)	0.30 (0.09–1.03)	NA	NA
Race (Black)*Education				
Graduated from high school	NA	3.61 (0.87–14.98)	NA	NA
One or more years of college	NA	6.82** (1.52–30.60)	NA	NA
Graduated from trade school	NA	10.32* (1.09–97.89)	NA	NA
Graduated from college	NA	5.23* (1.34–20.44)	NA	NA
Race (Black)*Employment (Yes)	NA	1.18 (0.59–2.37)	NA	NA

Significance

* *p*<0.05

** *p*<0.01

*** *p*<0.001

AOR stands for Adjusted Odds Ratio

**Table 5:** Logistic regression on the association between race, education, and employment with exposure to tobacco advertisement across groups based on tobacco use

Variables	All (n=3,028)		Ever smoker (n=1,942)	Never smoker (n=1,086)
	Model 1	Model 2	Model 3	Model 4
	AOR (95% CI)	AOR (95% CI)	AOR (95% CI)	AOR (95% CI)
**Education**				
Graduated from high school	1.27 (0.78–2.05)	0.43 (0.11–1.61)	0.42 (0.04–4.16)	0.49 (0.09–2.59)
One or more years of college	1.16 (0.78–2.05)	0.26* (0.07–0.99)	1.25 (0.02–2.60)	0.29 (0.05–1.61)
Graduated from trade school	2.05 (0.77–5.44)	0.33 (0.05–2.18)	0.26 (0.01–4.53)	0.42 (0.03–6.00)
Graduated from college	0.62 (0.38–1.02)	0.17** (0.05–0.60)	0.12 (0.01–1.06)	0.25 (0.05–1.17)
Employment (Yes)	1.07 (0.75–1.52	0.98 (0.56–1.69)	0.95 (0.01–1.06)	1.03 (0.53–1.97)
Ever smoker (Yes)	2.09** (1.34–3.24)	2.19** (1.41–3.41)	NA	NA
Current smoker (Yes)	2.07** (1.23–3.49)	2.01** (1.19–3.40)	2.13** (1.23–3.72)	NA
**Age**				
30 – 39	0.56* (0.36–0.87)	0.54** (0.34–0.84)	0.61 (0.23–1.63)	0.50* (0.30–0.83)
40 – 49	0.77 (0.47–1.27)	0.75 (0.45–1.24)	0.82 (0.31–2.18)	0.71 (0.38–1.32)
50 – 59	0.43*** (0.27–0.68)	0.41*** (0.26–0.66)	0.39* (0.16–0.94)	0.47* (0.25–0.88)
60 & more	0.29*** (0.18–0.46)	0.28*** (0.17–0.45)	0.27** (0.11–0.67)	0.29*** (0.16–0.54)
Gender (Men)	1.62** (1.18–2.23)	1.66** (1.21–2.29)	1.35 (0.81–2.23)	1.93** (1.26–2.95)
Race (Black)	1.41* (1.02–1.94)	0.30 (0.09–1.03)	0.20 (0.03–1.59)	0.45 (0.09–2.26)
Race (Black)*Education				
Graduated from high school	NA	3.61 (0.87–14.98)	3.33 (0.30–36.50)	3.59 (0.56–22.86)
One or more years of college	NA	6.82** (1.52–30.60)	5.58 (0.45–69.36)	7.00* (1.01–48.75)
Graduated from trade school	NA	10.32* (1.09–97.89)	7.49 (0.30–187.50)	15.92 (0.54–470.37)
Graduated from college	NA	5.23* (1.34–20.44)	6.81 (0.64–72.87)	3.97 (0.69–22.77)
Race (Black)*Employment (Yes)	NA	1.18 (0.59–2.37)	1.13 (0.33–3.91)	1.12 (0.46–2.72)

Significance

* *p*<0.05

** *p*<0.01

*** *p*<0.001

AOR stands for Adjusted Odds Ratio
